# Scaling of average receiving time on weighted polymer networks with some topological properties

**DOI:** 10.1038/s41598-017-02036-0

**Published:** 2017-05-18

**Authors:** Dandan Ye, Song Liu, Jia Li, Fei Zhang, Changling Han, Wei Chen, Yingze Zhang

**Affiliations:** 1 Institute of orthopaedics, the Third Hospital of Hebei Medical University, Shijiazhuang, Hebei 050051 P.R. China; 2 Key laboratory of biomechanics of Hebei Province, Shijiazhuang, Hebei 050051 P.R. China

## Abstract

In this paper, a family of the weighted polymer networks is introduced depending on the number of copies *f* and a weight factor *r*. The topological properties of weighted polymer networks can be completely analytically characterized in terms of the involved parameters and/or of the fractal dimension. Moreover, assuming that the walker, at each step, starting from its current node, moves to any of its neighbors with probability proportional to the weight of edge linking them, namely weight-dependent walk. Then, we calculate the average receiving time (ART) with weighted-dependent walks, which is the sum of mean first-passage times (MFPTs) for all nodes absorpt at the trap located at the central node as a recursive relation. The obtained remarkable results display that when $\frac{1}{f+1} < r < 1$, the ART grows sublinearly with the network size; when $r=\frac{1}{f+1}$, ART grows with increasing size *N*_*g*_ as ${\mathrm{ln}}^{2}{N}_{g}$; when $0 < r < \frac{1}{f+1}$, ART grows with increasing size *N*_*g*_ as ln *N*_*g*_. In the treelike polymer networks, ART grows with linearly with the network size *N*_*g*_ when *r* = 1. Thus, the weighted polymer networks are more efficient than treelike polymer networks in receiving information.

## Introduction

Complex networks, as a powerful tool to describe and characterize the natural and man-made systems, have attracted considerable attention in many fields, such as mathematics, biology, life science and engineering disciplines^1–3^. Besides, as rapid developing discipline, polymer science has attracted much attention in the past few years, since it provides a powerful tool to study the macromolecules with various structures^4^. Flexible polymer structures are various, such as dendrimers^5^, mesh-like polymers^6, 7^, fractals^8, 9^, dendritic^10, 11^, regular hyperbranched structures^12, 13^, scale-free and small-world networks^14, 15^, and so on.

Weighted networks represent the natural framework to describe natural, social, and technological systems, in which the intensity of a relation or the traffic between elements is an important parameters^16, 17^. In general terms, weighted networks are extension of networks or graphs^18, 19^, in which each edge between nodes *i* and *j* is associated with a variable *w*_*ij*_, called the weight. Much attention has been paid to the study of weighted networks because most real networks, which include airport networks^20^, ecosystems^21^, the Internet networks^22^ and so on, often show weighted properties, so it is also meaningful to investigate the behavior on the weighted networks^23^. Motivated by complex networks and polymer structures, Zhang *et al*. defined a category of treelike polymer networks controlled by a parameter, which is built in an iterative way^24, 25^. Combining the weighted networks^26^ and polymer structures, a family of the weighted polymer networks is introduced depending on the number of copies *f* and a weight factor *r*.

In 2015, Dai *et al*. introduced comprehensively three kinds of walks: random walk, weigh-dependent walk and strength-dependent walk on the weighted networks^27^. On weighted networks, the walker will choose an edge according to its weight of the node connected by it, i.e. weight-dependent walk. A key quantity related to weighted networks is the mean first-passage time(MFPT), that is, the expected first time for the walker starting from a source node to a given target node. The average receiving time (ART) is the sum of mean first-passage times (MFPTs) for all nodes absorpt at the trap located at a given target node^28–31^.

In this paper, we define a family of weighted polymer networks controlled by two parameters, which is built in an iterative way. According to the construction, we study some structural properties of the weighted polymer networks, showing that (1) in the limit of large network order *g*, the average degree of weighted polymer networks tends to 2; (2) when 0 < *r* < 1, their average node strength goes to zero as *g* increases; (3) their node strength distribution follows a power-law distribution; (4) the weighted polymer networks networks have small-world property: in the infinite network, the average weighted shortest path (AWSP) tends to be a constant value which depends on two parameters *f*, *r*. However, the average shortest path (ASP) increases logarithmically with the network size. Then, by applying recursive relations of weighted polymer networks, we calculate the average receiving time (ART) with weighted-dependent walks, which is the sum of mean first-passage times (MFPTs) for all nodes absorpt at the trap located at the central node. So we derive exactly the ART formula, which displays that in large networks, the leading behaviors of ART for the weighted polymer networks follow distinct scalings, with the trapping efficiency associated with the network size *N*_*g*_, the number of copies *f* and a weight factor *r*.

This paper is organized as follow. Based on weighted networks^26^ and polymer structures, a family of the weighted polymer networks is introduced depending on the number of copies *f* and a weight factor *r* in the next section. In Section 3, some a priori prescribed topology is described in terms of average degree, average node strength, node strength distribution, and the average weighted shortest path, depending on the two main parameters: the number of copies *f* and the weight factor *r*. In Section 4, the average receiving time (ART) with weighted-dependent walk is obtained by recursive formulas for *F*_1_(*g*) and *T*_*tot*_(*g*). In the last section, we draw some conclusions that (1) the topology of weighted polymer networks can be completely analytically characterized in terms of the involved parameters and/or of the fractal dimension; (2) the weighted polymer networks are more efficient than treelike polymer networks in term of receiving information.

## Weighted treelike networks

In this section, a family of weighted polymer networks are introduced. Intuited by polymer networks^24, 25^ and Weighted Fractal Networks (WFN for short)^26^, a family of weighted polymer networks are constructed in a deterministically iterative way.

Let r(0 < r < 1) be a positive real numbers, and f(f  ≥ 1) be a positive integer. Denote by *G*_*g*_ the weighted polymer networks after *g* iterations, and the following is the iterative algorithm to create weighted polymer networks:

1. For *g* = 0, *G*_0_ consists of an isolated node, called the central node. For *g* = 1, *f* new nodes are generated connecting the central node to form *G*_1_. Let *G*_1_ be our base graph, composed by *f* + 1 nodes and *f* edges with unit weight. The *f* + 1 nodes in *G*_1_ are all the attaching nodes, labeled by $0,1,2,\cdots ,f$.
2. For *g* = 2, *G*_2_ is obtained from *G*_1_: Let ${G}_{1}^{\mathrm{(0)}},{G}_{1}^{\mathrm{(1)}},\cdots ,{G}_{1}^{(f)}$ be *f* + 1 replicas of *G*_1_, whose weighted edges have been scaled by the weight factor *r*. For $i=0,1,2,\cdots ,f$, let us denote by *i*′ the central node in ${G}_{1}^{(i)}$. Then merge the central node *i*′ in ${G}_{1}^{(i)}$ and Node *i* in *G*_1_ into a single new node, still labelled by $i(i=0,1,\cdots ,f)$. Figure 1 illustrates the iterative construction processes of a particular network from *g* = 1 to *g* = 3 for the case of *f* = 3.

**Figure 1:**
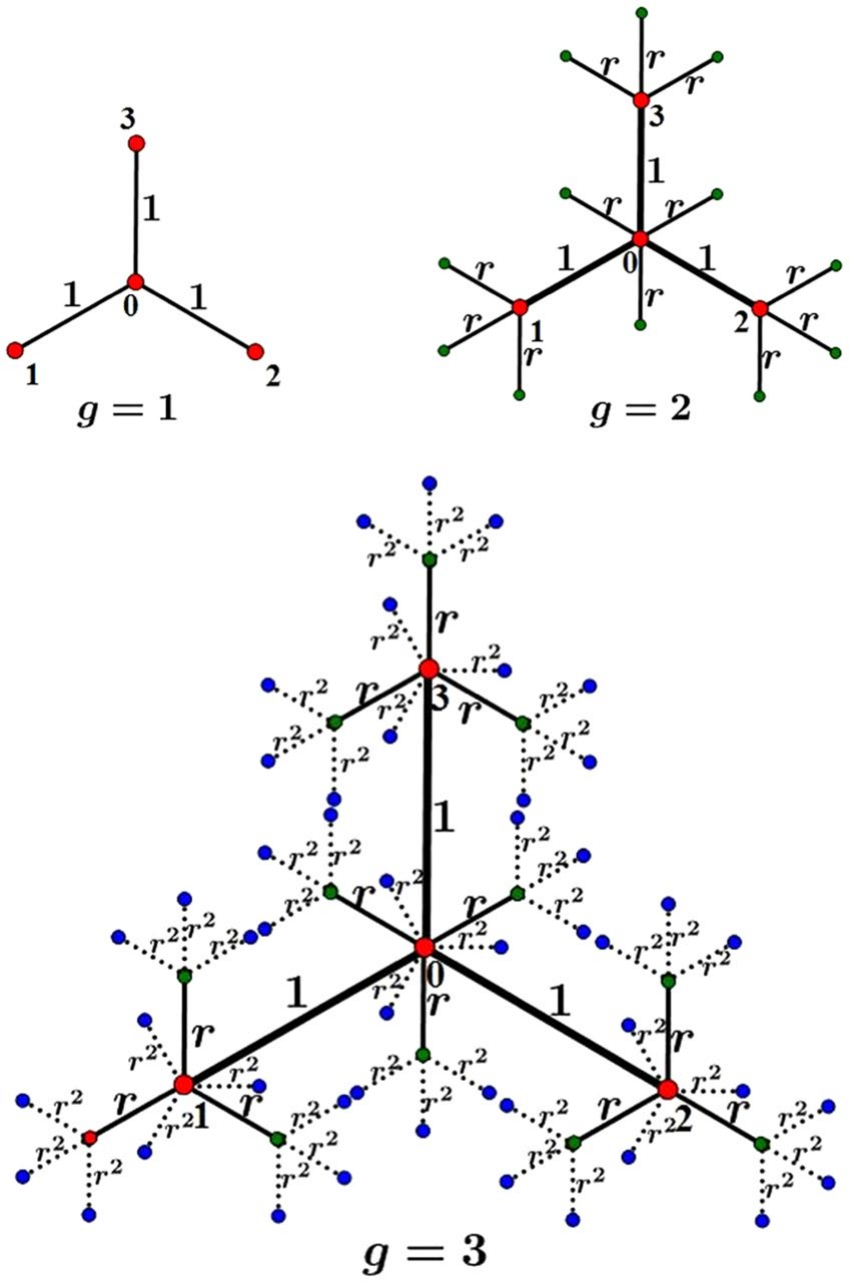
Iterative construction method for weighted polymer networks from *g* = 1 to *g* = 3 for the case of *f* = 3.
3. For *g* ≥ 1, *G*_*g*_ is obtained from *G*_*g*−1_ (see Fig. 2): Let ${G}_{g-1}^{(0)},{G}_{g-1}^{(1)},\cdots ,{G}_{g-1}^{(f)}$ be *f* + 1 replicas of *G*_*g*−1_, whose weighted edges have been scaled by the weight factor *r*. For $i=0,1,2,\cdots ,f$, let us denote by *i*′ the central node in ${G}_{g-1}^{(i)}$. Then merge the central node *i*′ in ${G}_{g-1}^{(i)}$ and Node *i* in *G*_1_ into a single new node, still labelled by $i(i=0,1,\cdots ,f)$. The weighted polymer networks is set up.

**Figure 2:**
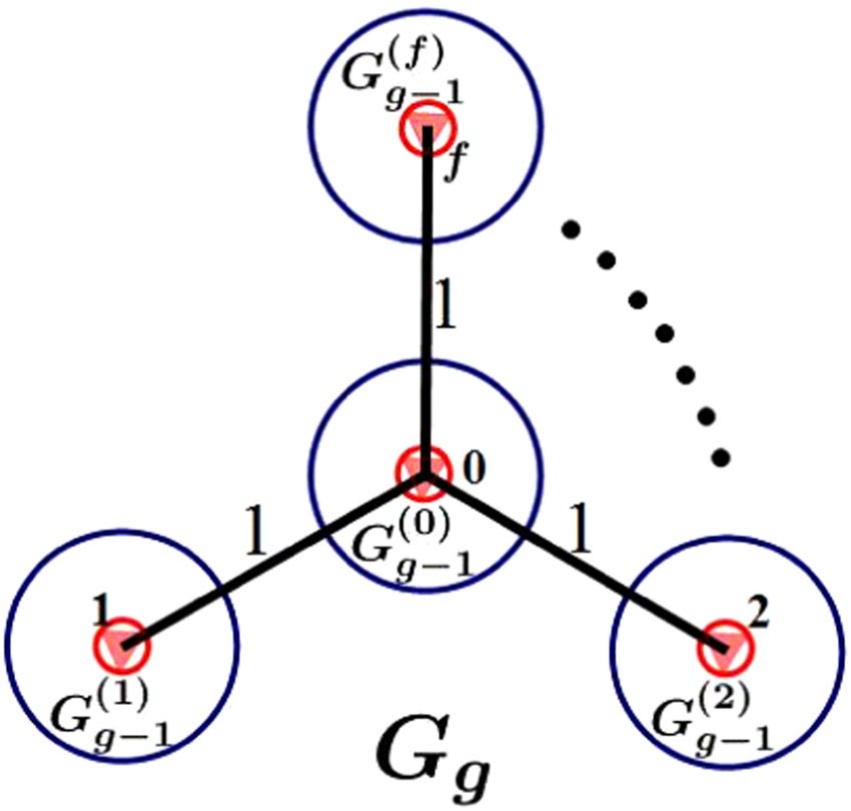
Construction method of weighted polymer networks. The open circles and triangles represent Node *i*′ of ${G}_{g-1}^{(i)}$ and *i* of ${G}_{1}(i=0,1,2,\cdots ,f)$, respectively.

The weighted polymer networks is one of type of WFN. According to Carletti and Righi^26^, WFN are scale-free, the exponent being the fractal dimension. WFN exhibit the “small-world” property (i.e. slow (logarithmic) increase of the average shortest path with the network size) and large average clustering coefficient. Thus, the fractal dimension of weighted polymer networks is completely characterized by two main parameters: the number of copies *f* ≥ 1 and the weight factor 0 < *r* < 1. We have that the fractal dimension of the weighted polymer networks is ${d}_{fract}=-\,\frac{\mathrm{log}(f+1)}{\mathrm{log}\,r}$.

According to the construction approach, it is easy to derive that at each iterative step *g*_*i*_(*g*_*i*_ ≥ 1), the number of newly generated nodes is $L({g}_{i})=f{(f+1)}^{{g}_{i}-1}$. Then the total number of nodes at each generation *g* is

1 $${N}_{g}=1+\sum _{{g}_{i}=1}^{g}L({g}_{i})={(f+1)}^{g},$$

and the total number of edges in *G*_*g*_ is *E*_*g*_ = *N*_*g*_ − 1 = (*f* + 1)^*g*^ − 1.

## Topological properties of weighted polymer networks

The aim of this section is to characterize the topology of weighted polymer networks, by analytically studying their properties such as the average degree, the average node strength, the node strength distribution, and the average weighted shortest path.

### Average degree and average node strength

The degree of a node *i* in a network, that is, the number of connections or edges the node *i* has to other nodes, is denoted by *deg*(*i*). The average degree of the weighted polymer networks *G*_*g*_, denoted by *ad*(*G*_*g*_), is defined as $ad({G}_{g})=\frac{2{E}_{g}}{{N}_{g}}$ ^32^. Hence in the limit of large *g*, the average degree *ad*(*G*_*g*_) is finite and it is asymptotically given by

$$ad({G}_{g})=\frac{2{E}_{g}}{{N}_{g}}=\frac{2{(f+1)}^{g}-2}{{(f+1)}^{g}}\to 2,\,g\to \infty .$$

In the weighted polymer networks *G*_*g*_, a weight *w*_*ij*_ is assigned to the edge connecting the nodes *i* and *j*, and the strength of node *i* can be defined as

2 $${s}_{i}=\sum _{j\in \nu (i)}{w}_{ij},$$

where the sum index *j* runs over the set *ν*(*i*) of neighbors of *i*. The strength of a node integrates the information concerning its connectivity and the weights of its links^33, 34^. Then using the recursive construction, we can explicitly compute the total node strength ${S}_{g}={\sum }_{i\in {G}_{g}}{s}_{i}$, and, provided $r\ne \frac{1}{f+1}$, easily show that

$${S}_{g}=2f\frac{{(fr+r)}^{g}-1}{fr+r-1}.$$

Because *r* < 1, we trivially find that the average node strength goes to zero as *g* increases: ${S}_{g}/{N}_{g}=\frac{2f{r}^{g}}{fr+r-1}-\frac{2f}{(fr+r-1){(f+1)}^{g}}\to 0(g\to \infty )$.

Especially, let us observe that the same is true if $r=\frac{1}{f+1}$; in this case, in fact *S*_*g*_ = 2*fg* grows linearly with *g*, thus slower than *N*_*g*_.

### Node strength distribution

Let *n*(*s*) denote the number of nodes in the weighted polymer networks *G*_*g*_ that have strength *s*. Let *s*_*i*_(*g*_*i*_) be the strength of any one of newly generated nodes *i* at each iterative step *g*_*i*_(*g*_*i*_ > 0). Assume that node *i* entered the networks at generation *g*(*g* > 0), then *s*_*i*_(*g*) = *r* ^*g*−1^. By construction, the strength of node *i* entered the networks at generation *g*_*i*_(0 < *g*_*i*_ < *g*) is ${s}_{i}({g}_{i})={r}^{{g}_{i}-1}+f({r}^{g-1}+{r}^{g-2}+\cdots +{r}^{{g}_{i}})$. For *g*_*i*_ = 0, the strength of the initial central node labeled by 0, equals to ${s}_{0}(0)=f({r}^{g-1}+{r}^{g-2}+\cdots +r+1)$. Using the property of iterative construction method, we can conclude:

$$n({s}_{i}({g}_{i}))=f{(f+1)}^{{g}_{i}-1},\,and\,\,n({s}_{0}(0))=1.$$

**Figure 3:**
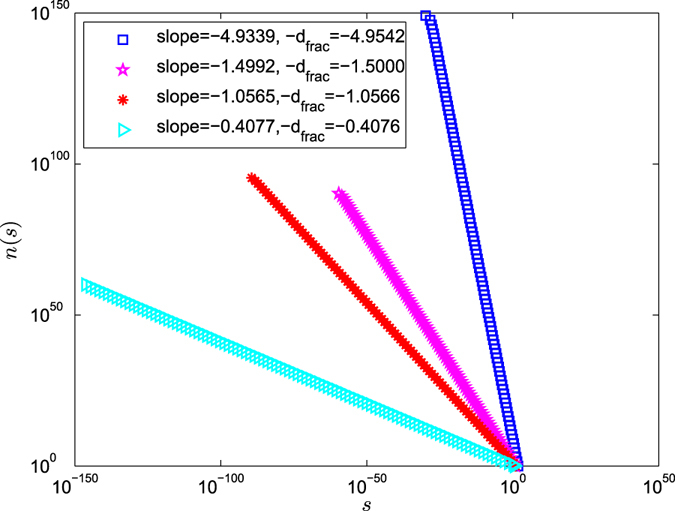
The log-log plot of *n*(*s*) versus *s* for different weight factor *r* and different copy number *f*.

For *g* = 100, *n*(*s*) versus *s* in the weighted polymer networks is on a log-log scale in Fig. 3. The slope of the weighted polymer networks *G*_100_ with 8^100^ nodes, *f* = 7 and *r* = 1/4 is −1.4992, which differs by 0.0008 from its the negative of the fractal dimension $-{d}_{frac}=\frac{\mathrm{log}\,8}{\mathrm{log}\,1\,/\,4}=-\,1.5000$. The slope of the weighted polymer networks *G*_100_ with 9^100^ nodes, *f* = 8 and *r* = 1/8 is −1.0565, which differs by 0.0001 from its the negative of the fractal dimension $-{d}_{frac}=\frac{\mathrm{log}\,9}{\mathrm{log}\,1\,/\,8}=-\,1.0566$. The slope of the weighted polymer networks *G*_100_ with 31^100^ nodes, *f* = 30 and *r* = 1/2 is −4.9339, which differs by 0.0203 from its the negative of the fractal dimension $-{d}_{frac}=\frac{\mathrm{log}\,31}{\mathrm{log}\,1\,/\,2}=-\,4.9542$. The slope of the weighted polymer networks *G*_100_ with 4^100^ nodes, *f* = 3 and *r* = 1/30 is −0.4077, which differs by −0.0001 from its the negative of the fractal dimension $-{d}_{frac}=\frac{\mathrm{log}\,4}{\mathrm{log}\,1\,/\,30}=-\,0.4076$. The results show that for different weight factor *r* and different copy number *f*, every line slope of log *n*(*s*) versus log *s* is nearly equal to the negative of the fractal dimension $-{d}_{fract}=\frac{\mathrm{log}(f+1)}{\mathrm{log}\,r}(0 < r < 1)$. This implies that *n*(*s*) are distributed according to a power law with exponent ${d}_{fract}=-\frac{\mathrm{log}(f+1)}{\mathrm{log}\,r}$. And therefore, for large *g*, *n*(*s*) can be obtained as

$$n(s)\sim c{s}^{-{d}_{fract}},$$

where *c* is constant. Thus, *n*(*s*) also follows a power-law distribution.

### Average weighted shortest path

By definition the average weighted shortest path(AWSP) of the weighted networks *G*_*g*_^35^ is given by

3 $$\overline{{d}_{g}}=\frac{2}{{N}_{g}({N}_{g}-1)}{S}_{tot}(g),$$

where

4 $${S}_{tot}(g)=\sum _{i,j\in {G}_{g},i\ne j}{d}_{ij}(g),$$

*d*_*ij*_(*g*) being the weighted shortest path linking nodes *i* and *j* in *G*_*g*_.

The modular recursive construction of *G*_*g*_ allows us to calculate the exact value of *S*_*tot*_(*g*). At step *g* + 1, we incise *G*_*g*_ into *f* + 1 branches, which we label as ${G}_{g}^{(i)}(i=0,1,\cdots ,f)$. Each branch ${G}_{g}^{(i)}(i=0,1,\cdots ,f)$ is a copy of *G*_*g*_ and has the same structure as *G*_*g*_, while their edge weights have been scaled by a weight factor r. The central nodes *i*′ of ${G}_{g}^{(i)}(i=1,\cdots ,f)$ are all connected to central node 0′ of ${G}_{g}^{(0)}$ by *f* edges with unit weight. Thus, the total of shortest distances *S*_*tot*_(*g*) satisfies the following recursion:

5 $${S}_{tot}(g+1)=(f+1)r{S}_{tot}(g)+{{\rm{\Omega }}}_{g},$$

where Ω_*g*_ is the sum over all weighted shortest paths whose nodes are not in the same copy of ${G}_{g}^{(i)}(i=0,1,\cdots ,f)$. Note that the weighted paths that contribute to Ω_*g*_ must all go through central node 0′ of ${G}_{g}^{(0)}$ at which the different ${G}_{g}^{(i)}(i=0,1,\cdots ,f)$ branches are connected. This recursive relation can be elaborated as follows:

The first term on the rhs of (5) describes the sum of the weighted shortest path linking nodes *i* and *j* in ${G}_{g}^{(i)}(i=0,1,\cdots ,f)$, respectively, i.e.,

$$\sum _{i,j\in {G}_{g}^{(0)}}+\sum _{i,j\in {G}_{g}^{(1)}}+\cdots +\sum _{i,j\in {G}_{g}^{(f)}}$$

Using the scaling mechanism for the edges, the above sum can be easily identified with

$$r\sum _{i,j\in {G}_{g}}+r\sum _{i,j\in {G}_{g}}+\cdots +r\sum _{i,j\in {G}_{g}}=(f+1)r\sum _{i,j\in {G}_{g}}=(f+1)r{S}_{tot}(g)$$

One can prove (see Method) that

6 $${{\rm{\Omega }}}_{g}=\left\{\begin{array}{lll}\frac{{f}^{2}}{r-1}{r}^{g}{(f+1)}^{2g}+\frac{{f}^{2}(r-2)}{r-1}{(f+1)}^{2g}, & if & 0 < r < 1,\\ {f}^{2}(g+1){(f+1)}^{2g}, & if & r=1.\end{array}\right.$$

Considering *S*_*tot*_(1) = *f* ^2^, we can solve Eq. (4) recursively to yield

7 $${S}_{tot}(g)=\left\{\begin{array}{lll}\frac{{f}^{2}(r-2)}{(f+1-r)(r-1)}{(f+1)}^{2g-1}+\frac{f(fr-1)}{(f+1)(r-1)}{r}^{g-1}{(f+1)}^{g} &  & \\ +(\frac{f}{r-1}-\frac{{f}^{2}(r-2)}{(f+1-r)(r-1)}){r}^{g-1}{(f+1)}^{2g-1}, & if & 0 < r < 1,\\ (fg-1){(f+1)}^{2g-1}+{(f+1)}^{g-1}, & if & r=1.\end{array}\right.$$

**Figure 4:**
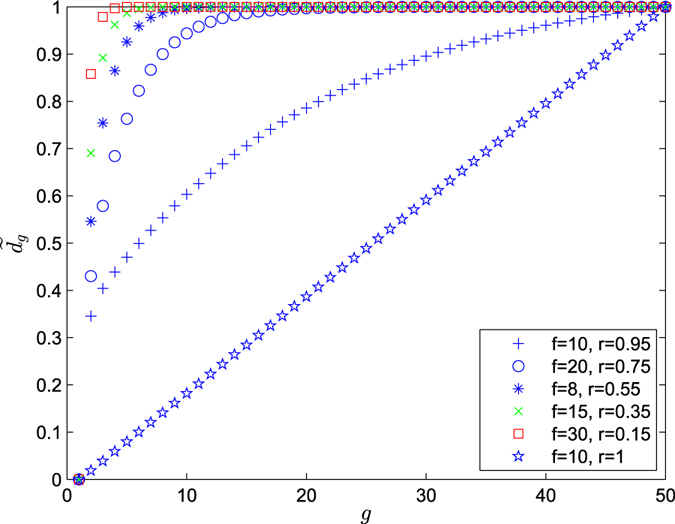
The average weighted shortest path. Plot of the renormalized average weighted shortest path $\tilde{{d}_{g}}$ versus the iteration *g*, where $\tilde{{d}_{g}}=\frac{\overline{{d}_{g}}-\,\min \,\{\overline{{d}_{g}}\}}{\max \,\{\overline{{d}_{g}}\}-\,\min \,\{\overline{{d}_{g}}\}}$.

We find that if *r* = 1 then *S*_*tot*_(*g*) = (*fg* − 1)(*f* + 1)^2*g*−1^ + (*f* + 1)^*g*−1^, which coincides with the *S*_*tot*_(*g*) in ref. 24. Therefore

8 $$\overline{{d}_{g}}=\left\{\begin{array}{lll}\frac{2{f}^{2}(r-2)}{(f+1-r)(r-1)}\frac{{(f+1)}^{g-1}}{{(f+1)}^{g}-1}+\frac{2f(fr-1)}{(f+1)(r-1)}\frac{{r}^{g-1}}{{(f+1)}^{g}-1} &  & \\ +2(\frac{f}{r-1}-\frac{{f}^{2}(r-2)}{(f+1-r)(r-1)})\frac{{r}^{g-1}{(f+1)}^{g-1}}{{(f+1)}^{g}-1}, & if & 0 < r < 1,\\ \frac{2(fg-1){(f+1)}^{g}+2}{{(f+1)}^{g+1}-(f+1)}, & if & r=1.\end{array}\right.$$

which provides the following asymptotic behavior in the limit of large *g* (see Fig. 4). When *g* → ∞,

9 $$\overline{{d}_{g}}\to \frac{2{f}^{2}(r-2)}{(f+1-r)(r-1)},\,if\,0 < r < 1$$

Thus the network grows unbounded but with the logarithm of the network size, while the weighted shortest distances stay bounded.

Recalling *N*_*g*_ = (*f* + 1)^*g*^ as given in Eq. (1), we have $g=\mathrm{ln}\,{N}_{g}/\mathrm{ln}(f+1)$. We can also compute the average shortest path (ASP), $\overline{{d}_{g}}$, formally obtained by setting *r* = 1. Hence, when the network size is large enough, we have

10 $$\overline{{d}_{g}}\cong \frac{2f}{(f+1)\,\mathrm{ln}(f+1)}\,\mathrm{ln}\,{N}_{g},\,if\,r=1.$$

## Average receiving time on weighted polymer networks

The purpose of this section is to determine explicitly the average receiving time 〈*T*〉_*g*_ and show how it scales with network order. We aim at a particular case on *G*_*g*_ with the perfect trap being located at the central node, labelled by 0. The process of biased walks is that the particle (walker), at each time step, starting from its current Node *i*, jumps to its neighbor Node *j* with probability ${p}_{i\to j}^{w}$ (see Eq. (11)).

For weight-dependent walk, a walker chooses one of its nearest neighbors with probability proportional to the weight of edge linking them^36, 37^. The transition probability from node *i* to its neighbor *j* is

11 $${p}_{i\to j}^{w}=\frac{{w}_{ij}}{{s}_{i}}=\frac{{w}_{ij}}{\sum _{j\in \nu (i)}{w}_{ij}},$$

where *s*_*i*_ denotes the strength of node *i* (see Eq. (2)).

For convenience of description, let us denote by $0,1,2,\cdots ,f$ the *f* + 1 attaching nodes in *G*_*g*_, and by $4,5,\cdots ,{N}_{g}-2$ and *N*_*g*_ − 1 all other nodes except for the *f* + 1 attaching nodes. Let *F*_*ij*_(*g*) be the mean first-passage time (MFPT) for a walker starting from Node *i* to Node *j*. Let *F*_*i*_(*g*) be the MFPT from Node *i* to the trap. 〈*T*〉_*g*_ is the average receiving time (ART), which is defined as the average of *F*_*i*_(*g*) over all starting nodes other than the trap. 〈*T*〉_*g*_ is the key question considered in this section.

By definition, 〈*T*〉_*g*_ is given by

$${\langle T\rangle }_{g}=\frac{1}{{N}_{g}}\sum _{i=0}^{{N}_{g}-1}{F}_{i}(g).$$

Here we denote by *T*_*tot*_(*g*) the sum of MFPTs for all nodes to absorption at the trap located the central Node 0, i.e.,

$${T}_{tot}(g)=\sum _{i=0}^{{N}_{g}-1}{F}_{i}(g).$$

Thus, the problem of determining 〈*T*〉_*g*_ is reduced to finding *T*_*tot*_(*g*). We will compute *T*_*tot*_(*g*) by segmenting *G*_*g*_.

From the iterative construction method of *G*_*g*_, *G*_*g*_ can be regarded as merging *f* + 1 groups, sequentially denoted by ${G}_{g-1}^{(i)}(i=0,1,\cdots ,f)$. The *f* + 1 groups are obtained as follows: ${G}_{g-1}^{(0)},{G}_{g-1}^{(1)},\cdots ,{G}_{g-1}^{(f)}$ are *f* + 1 replicas of *G*_*g*−1_, whose weighted edges have been scaled by the weight factor *r*. For $i=0,1,2,\cdots ,f$, let us denote by *i*′ the central node in ${G}_{g-1}^{(i)}$. Then merge the central node *i*′ in ${G}_{g-1}^{(i)}$ and Node *i* in *G*_1_ into a single new node, still labelled by $i(i=0,1,\cdots ,f)$. The process is described in Fig. 2.

Through this division we could rewrite the sum *T*_*tot*_(*g*) as follows:

12 $${T}_{tot}(g)=(f+1){T}_{tot}(g-1)+{N}_{g-1}\sum _{i=1}^{f}{F}_{i}(g).$$

We now elaborate Eq. (12). The first term on the rhs of Eq. (12) describes the sum of MFPTs for all nodes in ${G}_{g-1}^{(i)}$ to reach its attaching nodes $i(i=0,1,\cdots ,f)$. Recalling ${G}_{g-1}^{(i)}$ linked to Node $i(i=0,1,\cdots ,f)$ is a copy of *G*_*g*−1_ and the scaling mechanism for edges, the first term in the rhs of Eq. (12) can be identified with (*f* + 1)*T*_*tot*_(*g* − 1); the second term describes the sum of MFPTs for all nodes in ${G}_{g-1}^{(j)}$ from Node $j(j=1,2,\cdots ,f)$ to Central Node 0.

Because of the symmetry of nodes $1,2,\cdots ,f$, ${F}_{1}(g)={F}_{2}(g)=\cdots ={F}_{f}(g)$. Eq. (12) can be simplified as

13 $${T}_{tot}(g)=(f+1){T}_{tot}(g-1)+f{N}_{g-1}{F}_{1}(g).$$

Thus, the problem of determining *T*_*tot*_(*g*) is reduced to finding *F*_1_(*g*). Using the construction of the weighted polymer networks *G*_*g*_ and the scaling mechanism for edges, we obtain

14 $$\begin{array}{rcl}{F}_{1}(g) & = & (1+fr+f{r}^{2}+\cdots +f{r}^{g-1})+f[{r}^{g-1}{F}_{1}(1)+{r}^{g-2}{F}_{1}(2)\\  &  & +\cdots +{r}^{2}{F}_{1}(g-2)+r{F}_{1}(g-1)],\end{array}$$

and

15 $$\begin{array}{rcl}{F}_{1}(g-1) & = & (1+fr+f{r}^{2}+\cdots +f{r}^{g-2})+f[{r}^{g-2}{F}_{1}(1)\\  &  & +{r}^{g-3}{F}_{1}(2)+\cdots +{r}^{2}{F}_{1}(g-3)+r{F}_{1}(g-2)].\end{array}$$

From Eqs (14) and (15), we can further have

16 $${F}_{1}(g)-(fr+r){F}_{1}(g-1)=1+fr-r.$$

Considering the initial condition *F*_1_(1) = 1, we can solve recursively Eq. (16) to obtain

17 $${F}_{1}(g)=\left\{\begin{array}{lll}\frac{2fr{(fr+r)}^{g-1}}{fr+r-1}-\frac{fr+1-r}{fr+r-1}, & if & r\ne \frac{1}{f+1},\\ (1+fr-r)g-(fr-r), & if & r=\frac{1}{f+1}.\end{array}\right.$$

Considering *T*_*tot*_(1) = *f* and inserting Eq. (17), we can solve Eq. (13) inductively to yield

18 $$\begin{array}{rcl}{T}_{tot}(g) & = & \frac{2{f}^{2}{r}^{g+1}}{{(fr+r-1)}^{2}}{(f+1)}^{2g-1}-\frac{f(fr+1-r)}{fr+r-1}g{(f+1)}^{g-1}\\  &  & -\frac{2{f}^{2}r}{{(fr+r-1)}^{2}}{(f+1)}^{g-1},\,if\,r\ne \frac{1}{f+1},\\ {T}_{tot}(g) & = & \frac{f(1+fr-r)}{2}{g}^{2}{(f+1)}^{g-1}-\frac{f(1+3fr-3r)}{2}g{(f+1)}^{g-1}\\  &  & +f(1+fr-r){(f+1)}^{g-1},\,if\,r=\frac{1}{f+1}.\end{array}$$

Hence, 〈*T*〉_*g*_, which we are concerned about, could be expressed as follows:

19 $$\begin{array}{rcl}{\langle T\rangle }_{g} & = & \frac{2{f}^{2}r}{{(fr+r-1)}^{2}(f+1)}{(fr+r)}^{g}-\frac{f(fr+1-r)}{(fr+r-1)(f+1)}g\\  &  & -\frac{2{f}^{2}r}{{(fr+r-1)}^{2}(f+1)},\,if\,r\ne \frac{1}{f+1},\\ {\langle T\rangle }_{g} & = & \frac{f(1+fr-r)}{2(f+1)}{g}^{2}-\frac{f(1+3fr-3r)}{2(f+1)}g+\frac{f(1+fr-r)}{f+1},\,if\,r=\frac{1}{f+1}.\end{array}$$

We find that if *r* = 1 then

20 $${\langle T\rangle }_{g}=2{(f+1)}^{g-1}-\frac{(fg+2)}{f+1},$$

which coincides with the 〈*T*〉_*g*_ in ref. 24.

Recalling *N*_*g*_ = (*f* + 1)^*g*^ and *g* = ln *N*_*g*_/ln(*f* + 1), we have

21 $${\langle T\rangle }_{g}\approx \frac{2{N}_{g}}{f+1}-\frac{f\,\mathrm{ln}\,{N}_{g}}{(f+1)\,\mathrm{ln}(f+1)}\simeq \frac{2{N}_{g}}{f+1},\,if\,r=1,$$

which coincides with that in ref. 24.

For systems with large order, i.e. *N*_*g*_ → ∞,

22 $$\begin{array}{rcl}{\langle T\rangle }_{g} & \approx  & \frac{2{N}_{g}}{f+1},\,if\,r=1,\\ {\langle T\rangle }_{g} & \approx  & \frac{2{f}^{2}r}{{(fr+r-1)}^{2}(f+1)}{N}_{g}^{1+{\mathrm{log}}_{f+1}r}\\  & = & \frac{2{f}^{2}r}{{(fr+r-1)}^{2}(f+1)}{N}_{g}^{1-\frac{1}{{d}_{fract}}},\,if\,\frac{1}{f+1} < r < 1,\\ {\langle T\rangle }_{g} & \approx  & \frac{{f}^{2}}{{(f+1)}^{2}\,{\mathrm{ln}}^{2}(f+1)}\,{\mathrm{ln}}^{2}{N}_{g},\,if\,r=\frac{1}{f+1},\\ {\langle T\rangle }_{g} & \approx  & \frac{f(fr+1-r)}{(1-fr-r)(f+1)\,\mathrm{ln}(f+1)}\,\mathrm{ln}\,{N}_{g},\,if\,0 < r < \frac{1}{f+1}.\end{array}$$

**Figure 5:**
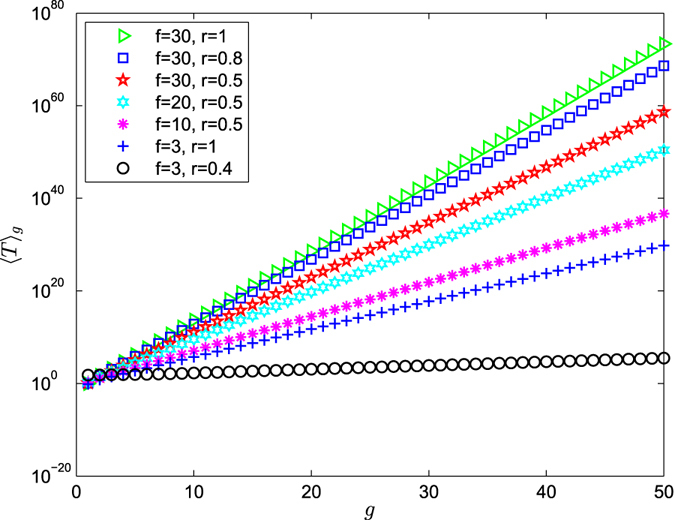
Average receiving time 〈*T*〉_*g*_ versus *g* is on a semilogarithmic scale for the range of $\frac{1}{f+1} < r\le 1$.

**Figure 6:**
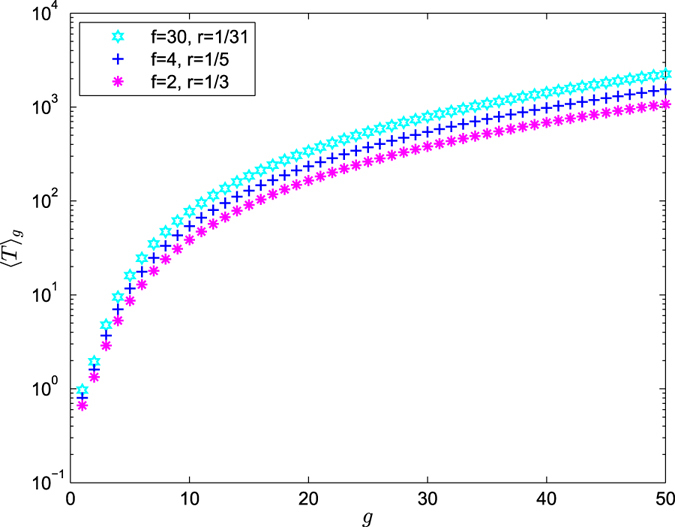
Average receiving time 〈*T*〉_*g*_ versus *g* is on a semilogarithmic scale for the range of $r=\frac{1}{f+1}$.

**Figure 7:**
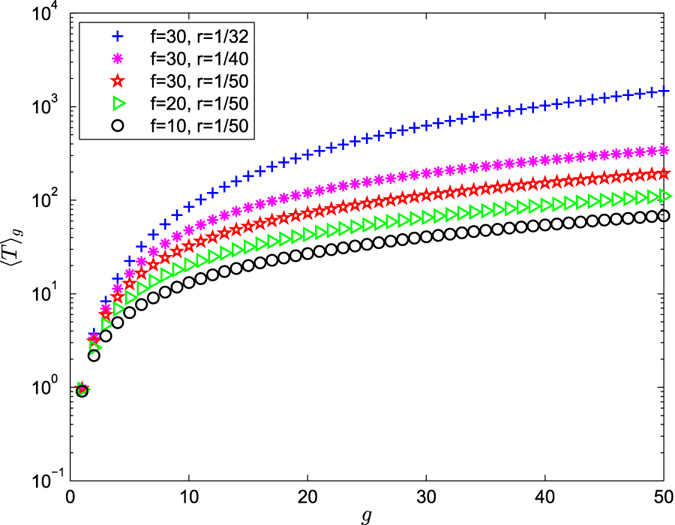
Average receiving time 〈*T*〉_*g*_ versus *g* is on a semilogarithmic scale for the range of $0 < r < \frac{1}{f+1}$.

According to Eqs (19) and (20), ART 〈*T*〉_*g*_ versus *g* for the range of *g* ≤ 50 on a semilogarithmic scale is shown in Figs 5, 6 and 7. From Eq. (22), we can have draw the conclusions as follows:

**Case 1**: $${\langle T\rangle }_{g}\approx \frac{2{N}_{g}}{f+1}$$.

When *r* = 1, ART grows linearly with the network size *N*_*g*_. Figure 5 shows that ART increases with the increase of the values of *f*. That is to say, the smaller the value of *f* is, the more efficient the trapping process is.

**Case 2**: $${\langle T\rangle }_{g}\approx \frac{2{f}^{2}r}{{(fr+r-1)}^{2}(f+1)}{N}_{g}^{1+{\mathrm{log}}_{f+1}r}=\frac{2{f}^{2}r}{{(fr+r-1)}^{2}(f+1)}{N}_{g}^{1-\frac{1}{{d}_{fract}}}$$.

When $\frac{1}{f+1} < r < 1$, in large network, the ART grows as a power-law function of the network size *N*_*g*_ with the exponent, represented by $\theta (f,r)=1+{\mathrm{log}}_{f+1}r=1-\frac{1}{{d}_{fract}}$ as follows:

1. When *f* is kept fixed, the exponent *θ*(*f*, *r*) is an increasing function of *r*(0 < *r* < 1) in Fig. 5. When *r* grows from 0 to 1, the exponent increases from 0 and approaches 1, indicating that ART grows sublinearly with the network size *N*_*g*_. This also means that the efficiency of the trapping process depends on the parameter *r*: the smaller the value of *r*, the more efficient the trapping process is.
2. When *r*(*r* ≠ 1) is kept fixed, ART grows sublinearly with the network size *N*_*g*_ and the exponent *θ*(*f*, *r*) is an increasing function of the values of *f* in Fig. 5. That is to say, the smaller the value of *f* is, the more efficient the trapping process is.
3. The fractal dimension *d*_*fract*_ of the weighted polymer networks play a relevant role in calculating the ART. The exponent $1-\frac{1}{{d}_{fract}}$ is an increasing function of the values of *d*_*fract*_, which means that the smaller the value of *d*_*fract*_ is, the more efficient the trapping process is.

**Case 3**: $${\langle T\rangle }_{g}\approx \frac{f\mathrm{(1}+fr-r)}{\mathrm{2(}f+\mathrm{1)}\,{\mathrm{ln}}^{2}(f+\mathrm{1)}}\,{\mathrm{ln}}^{2}{N}_{g}$$.

When $r=\frac{1}{f+1}$, ART grows with increasing size *N*_*g*_ as ln^2^ *N*_*g*_ according to Eq. (22). Figure 6 shows the smaller the value of *f* is, the more efficient the trapping process is.

**Case 4**: $${\langle T\rangle }_{g}\approx \frac{f(fr+1-r)}{(1-fr-r)(f+1)\,\mathrm{ln}(f+1)}\,\mathrm{ln}\,{N}_{g}$$.

When $0 < r < \frac{1}{f+1}$, ART grows with increasing size *N*_*g*_ as ln *N*_*g*_ in Fig. 7. When *f* is kept fixed, the smaller the value of *r*, the more efficient the trapping process is. When *r*(*r* ≠ 1) is kept fixed, the smaller the value of *f* is, the more efficient the trapping process is.

## Method

The analytical expression for Ω_*g*_ is not difficult to find, we denote as ${{\rm{\Omega }}}_{g}^{ab}$ the sum of all shortest paths with nodes in ${{\rm{\Omega }}}_{g}^{(a)}$ and ${{\rm{\Omega }}}_{g}^{(b)}$ $(a,b=0,1,2,\cdots ,f)$. Then the sum Ω_*g*_ is

$${{\rm{\Omega }}}_{g}={{\rm{\Omega }}}_{g}^{10}+{{\rm{\Omega }}}_{g}^{20}+\cdots +{{\rm{\Omega }}}_{g}^{f0}+{{\rm{\Omega }}}_{g}^{12}+{{\rm{\Omega }}}_{g}^{13}+\cdots +{{\rm{\Omega }}}_{g}^{1f}+{{\rm{\Omega }}}_{g}^{23}+{{\rm{\Omega }}}_{g}^{24}+\cdots +{{\rm{\Omega }}}_{g}^{25}+\cdots +{{\rm{\Omega }}}_{g}^{(f-1)f}.$$

By symmetry,

$${{\rm{\Omega }}}_{g}^{10}={{\rm{\Omega }}}_{g}^{20}=\cdots ={{\rm{\Omega }}}_{g}^{f0},$$

and

$${{\rm{\Omega }}}_{g}^{12}={{\rm{\Omega }}}_{g}^{13}=\cdots ={{\rm{\Omega }}}_{g}^{1f}={{\rm{\Omega }}}_{g}^{23}={{\rm{\Omega }}}_{g}^{24}=\cdots ={{\rm{\Omega }}}_{g}^{25}=\cdots ={{\rm{\Omega }}}_{g}^{(f-1)f},$$

so that

23 $${{\rm{\Omega }}}_{g}=f{{\rm{\Omega }}}_{g}^{10}+\frac{(f-1)f}{2}{{\rm{\Omega }}}_{g}^{12}.$$

In order to find ${{\rm{\Omega }}}_{g}^{10}$ and ${{\rm{\Omega }}}_{g}^{12}$, we define

$${{\rm{\Delta }}}_{g}=\sum _{i\in {G}_{g},i\ne 0}{d}_{i0}\mathrm{.}$$

We have

$${{\rm{\Delta }}}_{1}=\sum _{i=1}^{f}{d}_{i0}=f\mathrm{.}$$

Considering the self-similar network structure, we can easily know that at step *g*, the quantity Δ_*g*_ evolves recursively as

$${{\rm{\Delta }}}_{g}=r{{\rm{\Delta }}}_{g-1}+f(r{{\rm{\Delta }}}_{g-1}+{N}_{g-1})=(fr+r){{\rm{\Delta }}}_{g-1}+f{N}_{g-1}.$$

Using Δ_1_ = *f*, we have

$${{\rm{\Delta }}}_{g}={(fr+r)}^{g-1}{{\rm{\Delta }}}_{1}+f{(f+\mathrm{1)}}^{g-1}({r}^{g-2}+\cdots +r+\mathrm{1).}$$

If 0 < *r* < 1, then

$${{\rm{\Delta }}}_{g}=\frac{fr}{r-1}{(fr+r)}^{g-1}-\frac{f}{r-1}{(f+1)}^{g-1}.$$

If *r* = 1, then

$${{\rm{\Delta }}}_{g}=fg{(f+1)}^{g-1}.$$

On the other hand, we have

24 $$\begin{array}{rcl}{{\rm{\Omega }}}_{g}^{10} & = & \sum _{i\in {G}_{g}^{(1)}j\in {G}_{g}^{(0)}}{d}_{ij}\\  & = & \sum _{i\in {G}_{g}^{(1)}j\in {G}_{g}^{(0)}}({d}_{i1}+{d}_{10}+{d}_{j0})\\  & = & {N}_{g}\sum _{i\in {G}_{g}^{(1)}}{d}_{i1}+{N}_{g}^{2}+{N}_{g}\sum _{i\in {G}_{g}^{(0)}}{d}_{j0}\\  & = & {N}_{g}r{{\rm{\Delta }}}_{g}+{N}_{g}^{2}+{N}_{g}r{{\rm{\Delta }}}_{g}=2{N}_{g}r{{\rm{\Delta }}}_{g}+{N}_{g}^{2},\end{array}$$

25 $$\begin{array}{rcl}{{\rm{\Omega }}}_{g}^{12} & = & \sum _{i\in {G}_{g}^{(1)}j\in {G}_{g}^{(2)}}{d}_{ij}\\  & = & \sum _{i\in {G}_{g}^{(1)}j\in {G}_{g}^{(2)}}({d}_{i1}+{d}_{12}+{d}_{j2})\\  & = & {N}_{g}\sum _{i\in {G}_{g}^{(1)}}{d}_{i1}+2{N}_{g}^{2}+{N}_{g}\sum _{i\in {G}_{g}^{(2)}}{d}_{j2}\\  & = & {N}_{g}r{{\rm{\Delta }}}_{g}+2{N}_{g}^{2}+{N}_{g}r{{\rm{\Delta }}}_{g}=2{N}_{g}r{{\rm{\Delta }}}_{g}+2{N}_{g}^{2},\end{array}$$

where *d*_10_ = 1, *d*_12_ = 2 have been used.

Substituting Eqs (24) and (25) into Eq. (23), we have

$$\begin{array}{rcl}{{\rm{\Omega }}}_{g} & = & f(2{N}_{g}r{{\rm{\Delta }}}_{g}+{N}_{g}^{2})+\frac{f(f-1)}{2}(2{N}_{g}r{{\rm{\Delta }}}_{g}+2{N}_{g}^{2})\\  & = & fr(f+1){N}_{g}{{\rm{\Delta }}}_{g}+{f}^{2}{N}_{g}^{2}\end{array}$$

If 0 < *r* < 1, then

$${{\rm{\Omega }}}_{g}=\frac{{f}^{2}}{r-1}{r}^{g}{(f+1)}^{2g}+\frac{{f}^{2}(r-2)}{r-1}{(f+1)}^{2g}.$$

If *r* = 1, then

$${{\rm{\Omega }}}_{g}={f}^{2}(g+1){(f+1)}^{2g}.$$

## Conclusions

In this paper, we have introduced a family of weighted polymer networks, and studied its topological structure:(1) in the limit of large *g*, the average degree of weighted polymer networks tends to 2; (2) when 0 < *r* < 1, their average node strength goes to zero as *g* increases; (3) their node strength distribution follows a power-law distribution; (4) the weighted polymer networks networks have small-world property: in the infinite network, the AWSP tends to be a constant value which depends on two parameters *f*, *r*. However, the ASP increases logarithmically with the network size. Finally, we calculate the average receiving time (ART) with weighted-dependent walks on weighted polymer networks. Our analysis has indicated that (1) when $\frac{1}{f+1} < r < 1$, in large network, the ART grows as a power-law function of the network size *N*_*g*_ with the exponent, represented by $\theta (f,r)=1+{\mathrm{log}}_{f+1}r=1-\frac{1}{{d}_{fract}}$. ART grows sublinearly with the network size *N*_*g*_ and the exponent $1-\frac{1}{{d}_{fract}}$ is an increasing function of the values of *d*_*fract*_, which means that the smaller the value of *d*_*fract*_ is, the more efficient the trapping process is; (2) when $r=\frac{1}{f+1}$, ART grows with increasing size *N*_*g*_ as ln^2^ *N*_*g*_; (3) when $0 < r < \frac{1}{f+1}$, ART grows with increasing size *N*_*g*_ as ln *N*_*g*_. In the treelike polymer networks, ART grows with linearly with the network size *N*_*g*_ when *r* = 1. Thus, the weighted polymer networks are more efficient than treelike polymer networks in receiving information.
